# Factors Associated With Intraocular Inflammation in Neovascular Age-Related Macular Degeneration Patients Treated With Brolucizumab

**DOI:** 10.1167/iovs.65.1.8

**Published:** 2024-01-03

**Authors:** Yuto Hashimoto, Satoru Inoda, Hidenori Takahashi, Ryota Takahashi, Hana Yoshida, Yujiro Fujino, Shinichi Sakamoto, Hidetoshi Kawashima, Yasuo Yanagi

**Affiliations:** 1 Department of Ophthalmology, Jichi Medical University, Tochigi, Japan; 2 Department of Ophthalmology, Japan Community Healthcare Organization Tokyo Shinjuku Medical Center, Tokyo, Japan; 3 Department of Ophthalmology and Micro-Technology, Yokohama City University Medical Center, Yokohama, Japan; 4 The Ophthalmology & Visual Sciences Academic Clinical Program, Duke-NUS Medical School, National University of Singapore, Singapore

**Keywords:** brolucizumab, intraocular inflammation, aqueous humor, age-related macular degeneration

## Abstract

**Purpose:**

To identify factors associated with intraocular inflammation (IOI) in patients with neovascular age-related macular degeneration (nAMD) treated with brolucizumab.

**Methods:**

In this prospective observational study, we collected aqueous humor samples from 96 eyes of 96 patients receiving treatment with brolucizumab; IOI subsequently developed in 19 eyes of 19 patients. To identify cytokines upregulated in eyes with subsequent development of IOI, we compared the aqueous humor cytokine levels between the IOI and non-IOI groups. We also collected plasma from 20 patients who developed IOI and 20 age- and sex-matched controls to identify differences in plasma biomarkers and the subfraction of CD4^+^ cells. Using stepwise variable selection and multivariate binary regression analysis, we developed an algorithm that accurately assessed the likelihood of IOI occurrence.

**Results:**

The IOI group showed elevated aqueous humor levels of P-selectin (584 vs. 324 pg/mL, *P* = 0.013), TNF-α (0.89 vs. 0.60 pg/mL, *P* = 0.018), and IL-1α (2.0 vs. 1.4 pg/mL, *P* = 0.035) compared with the non-IOI group. Serum MMP-9 concentrations were higher in the IOI group than the non-IOI group (18,310 vs. 13,450 pg/mL, *P* = 0.029). Furthermore, the percentage of Th2 cells was significantly decreased in the IOI compared with the non-IOI group (3.1% vs. 4.2%, *P* = 0.013). The receiver operating characteristic curves for the optimal models showed an area under the curve ranging from 0.71 to 0.89, indicating good performance.

**Conclusions:**

The combination of elevated concentrations of multiple aqueous humor cytokines and of serum MMP-9 and a lower number of plasma Th2 cells is associated with brolucizumab-related IOI in patients with nAMD.

Brolucizumab is an anti-vascular endothelial growth factor (VEGF) drug with antibody variable domains for target binding, also known as humanized single-chain variable fragments.^1^ The high solubility of brolucizumab enables its intravitreous administration at high concentrations. Together with its small molecular size, which facilitates improved tissue penetration and bioavailability, brolucizumab exhibits longer durability and improved efficacy in eyes with neovascular age-related macular degeneration (nAMD) compared with other available treatments.^2^^–^^4^

However, in the phase III HAWK and HARRIER studies, aseptic intraocular inflammation (IOI), retinal vasculitis, and retinal vascular occlusion were observed at a greater rate after brolucizumab injections compared with aflibercept.^4^ Since its launch on the market, the frequency of IOI has been higher for brolucizumab than for other anti-VEGF drugs. The occurrence of IOI after brolucizumab may range from peripheral vasculitis to occlusion of large retinal arteries around the optic nerve or macula with severe vision loss.^5^

Retinal vasculitis and IOI after the intravitreal injection of brolucizumab are characterized by variable occlusion of large or small retinal arteries, or both, and perivenular abnormalities. As such, it has been hypothesized that IOI is due to a delayed-type hypersensitivity.^6^ Although some studies have attempted to identify the cause and underlying mechanisms of IOI, the risk factors still require elucidation.^7^^–^^16^

From June 2020 to December 2021, we administered brolucizumab to 390 eyes of 342 patients. Similar to previous reports in Asian countries, about 50% of new patients with nAMD at our institution are diagnosed with polypoidal choroidal vasculopathy (PCV).^17^ In the HAWK and HARRIER trials,^18^ PCV was treated as a subtype of nAMD, and we also use brolucizumab for PCV patients. Among those who received brolucizumab injections, aseptic IOI occurred in 31 eyes of 28 patients: seven eyes of seven patients had type 1 macular neovascularization (MNV), 21 eyes of 19 patients had PCV, and three eyes of two patients had type 3 MNV.

In our institution, we invite all patients with retinal pathology to join an institutional ethics committee-approved prospective clinical study in which we collect aqueous humor samples immediately before anti-VEGF injections and store them, as previously reported.^17^ In this study, only two of 390 nAMD patients who received brolucizumab injections declined to participate. This provided a unique opportunity to examine the differences in the pre-injection aqueous humor protein profiles of eyes that do and do not develop post-brolucizumab IOI and to establish a model to discriminate those who develop IOI based on the cytokine profile at the time of the first brolucizumab injection. For the current study, we also collected blood samples from IOI patients and 20 non-IOI patients matched for age, sex, and brolucizumab use as first-line therapy to examine the differences in blood markers between the two groups.

## Methods

### Study Design and Approval

We obtained samples from a prospective study that followed the tenets of the Declaration of Helsinki. All patients provided informed consent (UMIN000020718). Institutional review board approval was provided by Jichi Medical University (E12-86) and Japan Community Healthcare Organization Tokyo Shinjuku Medical Center (H22-8-9).

From June 2020 to December 2021, 390 eyes of 342 patients received brolucizumab at Jichi Medical University and Japan Community Healthcare Organization Tokyo Shinjuku Medical Center. When both eyes were treated with brolucizumab, we selected the eye with better visual acuity and excluded the other eye. From the medical records, we extracted age, sex, disease type, naïve patients, duration of therapy, number of injections before brolucizumab switch, last injection interval, history of photodynamic therapy, and visual acuity.

### Procedure

Multiplex analysis was performed for selected samples. Among all 31 IOI patients, the aqueous humor was analyzed in 19 eyes of 19 patients: 15 eyes of 15 AMD patients who developed IOI after switching from aflibercept to brolucizumab and four eyes of four nAMD patients who developed IOI after receiving brolucizumab as first-line treatment (IOI group). The non-IOI group was comprised of 63 eyes of 63 patients with nAMD who did not develop IOI after switching and 14 eyes of 14 patients with nAMD who did not develop IOI after receiving brolucizumab as first-line treatment. Samples for measurement were carefully selected to ensure that there were no significant differences in age, sex, interval between the last dose and the first dose before switching to brolucizumab, overall number of doses before switching to brolucizumab, and period between the date of the first dose of an anti-VEGF drug and the first brolucizumab dose. We defined IOI as iritis with an anterior chamber cell grading of ≥1+ using the Standardization of Uveitis Nomenclature grading system after brolucizumab and without signs of infectious IOI, such as acute worsening of vitreous opacity and hypopyon within several hours. We used 24 age-matched eyes of 24 patients without retinal disease who underwent cataract surgery as controls.

From the nAMD patients, we collected ∼0.2 mL aqueous humor immediately before brolucizumab injection. For the controls, we manually aspirated a sample of undiluted aqueous humor (usually ∼0.2 mL) into a disposable syringe at the beginning of cataract surgery, immediately transferred it to a sterile ProteoSave tube (Sumitomo Bakelite Co., Ltd., Tokyo, Japan), and stored it at −80°C until measurement.

For T helper (Th) cell analysis, we collected blood from 20 IOI patients (aqueous humor had been collected from 14 of these patients) and 20 non-IOI patients matched for age, sex, and brolucizumab use as first-line therapy at Jichi Medical University from December 2021 to February 2022. Blood was collected 111 days (SD, 102; range, 28–469) and 108 days (SD, 65; range, 49–287) after the last injection of any anti-VEGF drug in the non-IOI and IOI groups, respectively. From IOI patients, blood was collected 306 days (SD, 193; range, 35–581) after the onset of IOI. We also collected preoperative blood samples from 20 patients without retinal disease who underwent cataract surgery.

### Measurements of Ocular and Serum Proteins

We determined the concentrations of the following proteins using a multiplex cytokine assay kit (Human Luminex Discovery Assay [LXSAHM-24]; R&D Systems, Minneapolis, MN, USA): monocyte chemotactic protein 1 (MCP-1); MCP-3; chemokine ligand 1 (CXCL1); CXCL13; interferon-gamma (IFN-γ); IFN-γ–inducible protein-10 (IP-10); granulocyte colony-stimulating factor (G-CSF); granulocyte macrophage colony-stimulating factor (GM-CSF); interleukin (IL)-1α, IL-1β, IL-2, IL-5, IL-6, IL-8, IL-10, IL-12p70, IL-17A; matrix metalloproteinase 1 (MMP-1); MMP-9; intercellular adhesion molecule 1 (ICAM-1); E-selectin; P-selectin; tumor necrosis factor-alpha (TNF-α); and VEGF-A. Their detection limits were 9.9, 3.2, 5.3, 11.5, 0.4, 1.2, 4.1, 4.1, 0.9, 0.8, 1.8, 0.5, 1.7, 1.8, 1.6, 20.2, 1.8, 2.7, 13.6, 87.9, 18.8, 9.0, 1.2, and 2.1 pg/mL, respectively. We measured the aqueous humor samples without dilution, whereas the serum samples were diluted twofold before measurement. We performed the measurements twice for each sample and calculated the average value.

The fraction of CD4^+^ cells (Th cells) in plasma was evaluated using flow cytometry by a commercial clinical laboratory (SRL, Inc., Tokyo, Japan) according to the presence of IFN-γ and IL-4. The cells were classified as Th1 (IFN-γ^+^/IL-4^−^), Th2 (IFN-γ^−^/IL-4^+^), Th0 (IFN-γ^+^/IL-4^+^), or IFN-γ^−^/IL-4^−^, and their percentages were determined. The Th1-to-Th2 ratio was also calculated.

### Diagnosis of nAMD

We routinely perform fluorescein angiography in patients with nAMD to diagnose nAMD and to discriminate types 1, 2, and 3 MNV, except in patients with contraindications due to drug hypersensitivity, liver dysfunction, or recent cerebrovascular events.^19^ We performed indocyanine green angiography, together with fluorescein angiography, to identify PCV.^20^ We conducted swept-source optical coherence tomography using a Silverstone imaging device (Nikon, Tokyo, Japan) or DRI OCT Triton (Topcon, Tokyo, Japan), or spectral-domain optical coherence tomography using an RS-3000 Advance system (Nidek, Aichi, Japan) on each patient at each visit.

### Statistical Analysis

We performed statistical analysis using JMP Pro 16.2.0 (SAS Institute, Cary, NC, USA). We assessed categorical data using Pearson's χ^2^ tests and analyzed continuous variables using Student's *t*-tests with a two-tailed distribution after normalization to compare the presence and absence of post-brolucizumab IOI in nAMD patients and controls. Because the concentrations of all cytokines had log-normal distributions, we used log-transformed concentrations. We did not use Bonferroni's correction in univariate analysis because this was a hypothesis-generating exploratory study whose purpose was to identify a possible association between post-brolucizumab IOI and aqueous humor proteins. There were slight but non-significant differences in age and sex; accordingly, to ensure the robustness of the results, additional analyses were performed. First, we performed multivariate analysis using age and sex as explanatory variables. Subsequently, we used the mean values to adjust for age and sex and applied these adjustments to the entire sample. In the subsequent step, we performed *t*-tests using the adjusted dataset. Essentially, our approach closely resembled a multivariate analysis of variance (MANOVA).

To assess the occurrence of post-brolucizumab IOI, we used multivariate binary logistic regression analysis with an effect likelihood ratio test to compare the aqueous cytokine concentrations between patients with and without post-brolucizumab IOI. To create a reliable equation that estimates outcomes based on a limited number of cases, we applied stepwise variable selection (with the minimum corrected Akaike's information criterion to reduce the number of variables) to select the explanatory variables for protein concentrations^21^. The multivariate analysis included correction for multiple comparisons. We performed area under the receiver operating characteristic (ROC) curve (AUC) analysis to assess the discriminative power of the models and to calculate their sensitivity and specificity at the best threshold of the Youden index. We used only partially overlapping populations for this analysis. As such, we excluded blank values from each analysis.

## Results

### Characteristics of All Patients

Patient characteristics are shown in Table 1. There were 28 patients in the IOI group and 314 in the non-IOI group. The mean ages of the patients in the IOI and non-IOI groups were 78.1 years (SD, 9.1; range, 58–101) and 74.9 years (SD, 9.5; range, 48–121), respectively (*P* = 0.086). There were 15 male patients (54%) and 223 male patients (71%) in the IOI and non-IOI groups, respectively (*P* = 0.14). Patients received bilateral brolucizumab injection on the same day when IOI did not occur after the first unilateral injection. Among these 48 patients, IOI developed in three patients in both eyes. The groups were well controlled for all characteristics (Table 1).

**Table 1.** Characteristics of All Patients

Characteristic	IOI	Non-IOI	*P* vs. IOI
*n*	28	314	—
Age (y), mean (SD)	78.1 (9)	74.9 (10)	0.086^*^
Male sex, *n* (%)	15 (54)	223 (71)	0.14^†^
PCV, *n* (%)	19 (68)	160 (51)	0.13^†^
Typical nAMD (excluding RAP), *n* (%)	7 (25)	140 (45)	—
RAP, *n* (%)	2 (7)	14 (4)	—
Duration of therapy (mo), mean (SD)	38.1 (34)	53.3 (50)	0.31^*^
Number of injections, mean (SD)	10 (11)	9.2 (12)	0.70^*^
Last injection interval (wk), mean (SD)	10.1 (10)	17.5 (28)	0.18^*^
Naïve patients, *n* (%)	8 (29)	128 (41)	0.21^†^
History of PDT, *n* (%)	4 (14)	42 (13)	0.89^†^
Visual acuity (logMAR), mean (SD)	0.52 (0.38)	0.63 (0.36)	0.15^*^

PCV, polypoidal choroidal vasculopathy; PDT, photodynamic therapy; RAP, retinal angiomatous proliferation.

* Student's *t*-test.

† Pearson's χ^2^ test.

### Characteristics of the Patients Who Provided Aqueous Humor Samples

The characteristics of the patients from whom aqueous humor samples were collected are presented in Table 2. There were 19 patients in the IOI group, 77 patients in the non-IOI group, and 24 individuals in the control group. The mean ages of the patients in the IOI, non-IOI, and control groups were 79.1 years (SD, 10; range, 58–101), 75.8 years (SD, 6.9; range, 57–94; *P* = 0.096 vs. IOI), and 75.3 years (SD, 8.9; range, 60–88; *P* = 0.54 vs. all AMD), respectively. There were 11 males among the 19 patients (58%) in the IOI group, 48 males among the 77 patients (62%) in the non-IOI group (*P* = 0.72 vs. IOI), and 14 males among the 24 patients (58%) in the control group (*P* = 0.78 vs. all AMD). According to our protocol, the groups were largely well controlled for all characteristics (Table 2).

**Table 2.** Characteristics of Patients From Whom Aqueous Humor Samples Were Collected

Characteristic	IOI	Non-IOI	*P* vs. IOI	Control Cataract Eyes	*P* vs. AMD
*n*	19	77	—	24	—
Age (y), mean (SD)	79 (10)	76 (7)	0.096^*^	75 (9)	0.54^*^
Male sex, *n* (%)	11 (58)	48 (62)	0.72^†^	14 (58)	0.78^†^
Duration of therapy (mo), mean (SD)	34 (35)	44 (38)	0.31^*^	—	—
Number of injections, mean (SD)	10.8 (11)	13.2 (12)	0.43^*^	—	—
Naïve patients, *n* (%)	2 (22)	10 (19)	0.83^†^	—	—
Last injection interval (wk), mean (SD)	8 (5)	10 (6)	0.16^*^	—	—
PCV, *n* (%)	13 (68)	45 (58)	0.53^†^	—	—
Typical nAMD (excluding RAP), *n* (%)	5 (26)	30 (39)		—	—
RAP, *n* (%)	1 (5)	2 (3)		—	—
History of PDT, *n* (%)	3 (16)	13 (17)	0.91^†^	—	—

* Student's *t*-test.

† Pearson's χ^2^ test.

### Aqueous Humor Protein Concentrations in the IOI, Non-IOI, and Control Groups Before Brolucizumab Injection

**Figure 1.**
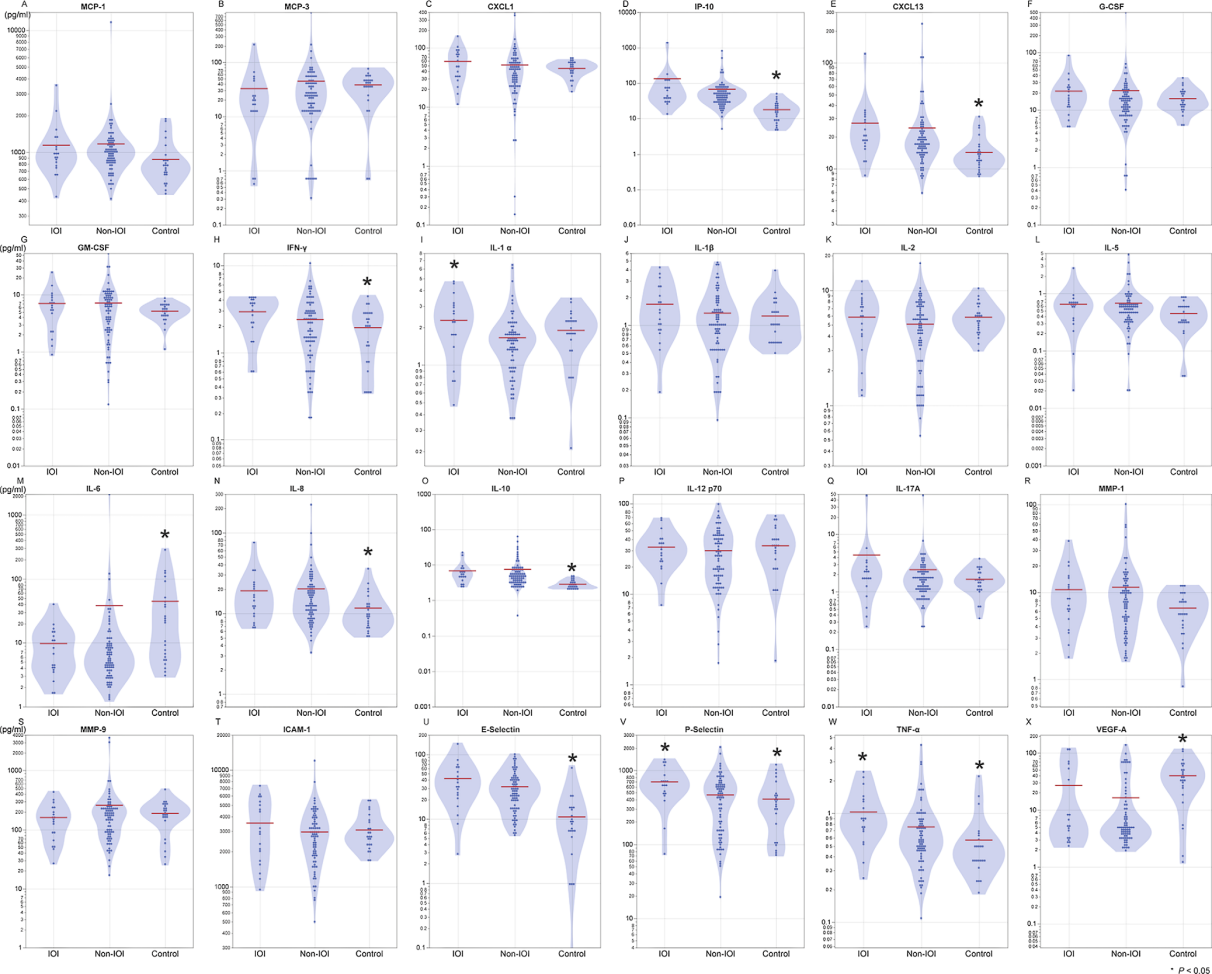
Protein concentrations in the aqueous humor of the IOI, non-IOI, and control groups before brolucizumab injection. *Error bar* indicates standard error. Comparisons between AMD patients and controls. The aqueous humor profiles of the IOI and non-IOI groups showed significant differences in the concentrations of P-selectin (584 vs. 324 pg/mL, respectively; *P* = 0.013), TNF-α (0.89 vs. 0.60 pg/mL; *P* = 0.018), and IL-1α (2.0 vs. 1.4 pg/mL; *P* = 0.035), whereas no significant differences were found in IFN-γ (2.5 vs. 1.8 pg/mL; *P* = 0.086), IP-10 (61 vs. 45 pg/mL; *P =* 0.15), CXCL1 (48 vs. 32 pg/mL; *P* = 0.16), IL-1β (1.3 vs. 1.0 pg/mL; *P* = 0.20), G-CSF (16 vs. 12 pg/mL; *P* = 0.21), IL-12p70 (29 vs. 23 pg/mL; *P* = 0.24), CXCL13 (22 vs. 19 pg/mL; *P =* 0.27), E-selectin (31 vs. 25 pg/mL; *P* = 0.27), or VEGF-A (10 vs. 7.4 pg/mL; *P* = 0.30).

Figure 1 shows the protein concentrations in the aqueous humor of the IOI, non-IOI, and control groups. Compared with the non-IOI group, the IOI group showed higher levels of P-selectin (584 vs. 324 pg/mL; *P* = 0.013), TNF-α (0.89 vs. 0.60 pg/mL; *P* = 0.018), and IL-1α (2.0 vs. 1.4 pg/mL; *P* = 0.035). To correct for a trivial and statistically insignificant difference in age and sex between the groups, adjustments were made for age and sex, as well as for age, sex, duration of therapy, axial length, and posterior vitreous detachment. The results revealed significant differences in the same cytokines between the IOI and non-IOI groups.

### Characteristics of the Patients From Whom Serum was Collected

The characteristics of the 20 IOI and 20 non-IOI patients who provided blood samples are shown in Table 3. The mean ages of the patients in the IOI and non-IOI groups were 78 ± 10 years and 76 ± 8 years, respectively (*P* = 0.53). There were 12 males among the 20 patients (60%) in each group. According to our protocol, the groups were largely well controlled for all characteristics (Table 3).

**Table 3.** Characteristics of Patients From Whom Blood Samples Were Collected

Characteristic	IOI	Non-IOI	*P* vs. IOI
*n*	20	20	
Age (y), mean (SD)	78 (10)	76 (8)	0.53^*^
Male sex, *n* (%)	12 (60)	12 (60)	1.0^†^
Duration of therapy (mo), mean (SD)	44 (34)	69 (44)	0.24^*^
Number of injections, mean (SD)	13 (12)	14 (12)	0.32^*^
Naïve patients, *n* (%)	3 (15)	5 (25)	0.43^†^
Last injection interval (wk), mean (SD)	11 (11)	14 (7)	0.70^*^
PCV, *n* (%)	15 (75)	10 (50)	0.23^†^
Typical neovascular AMD (excluding RAP), *n* (%)	4 (20)	9 (45)	—
RAP, *n* (%)	1 (5.0)	1 (5.0)	—
History of PDT, *n* (%)	3 (15)	5 (25)	0.43^†^

* Student's *t*-test.

† Pearson's χ^2^ test.

### Serum Protein Concentrations in the IOI and Non-IOI Groups

**Figure 2.**
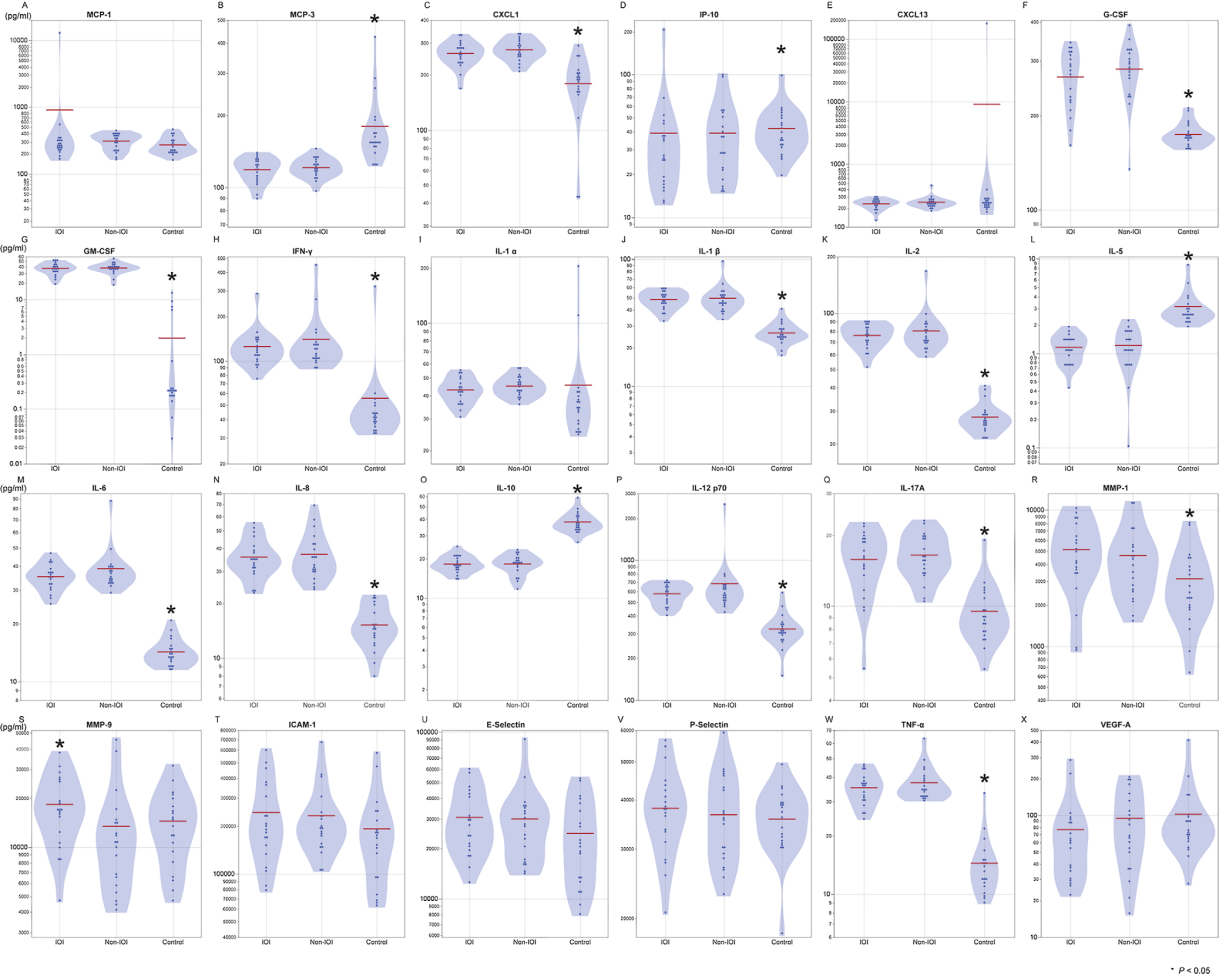
Protein concentrations in the sera of the IOI, non-IOI, and control groups. The serum concentration of MMP-9 differed significantly between the IOI and non-IOI patients (16,000 vs. 11,000 pg/mL, respectively; *P* = 0.029). No significant differences were evident between the groups in MCP-3 (63 vs. 72 pg/mL; *P* = 0.19), IL-6 (35 vs. 38 pg/mL; *P* = 0.24), IL-1α (43 vs. 45 pg/mL; *P* = 0.26), or CXCL1 (260 vs. 273 pg/mL; *P* = 0.30).

Figure 2 shows the serum protein concentrations in the IOI, non-IOI, and control groups. The concentrations of MMP-9 were higher in IOI patients than in non-IOI patients (18,310 vs. 13,450 pg/mL, *P* = 0.029). The graphed results, which were corrected for age and sex, as well as those corrected for age, sex, previous treatment, axial length, and posterior vitreous detachment, exhibited similarities to those of the uncorrected outcomes. This suggests that the corrections made for these factors did not significantly alter the overall trends in the data.

**Figure 3.**
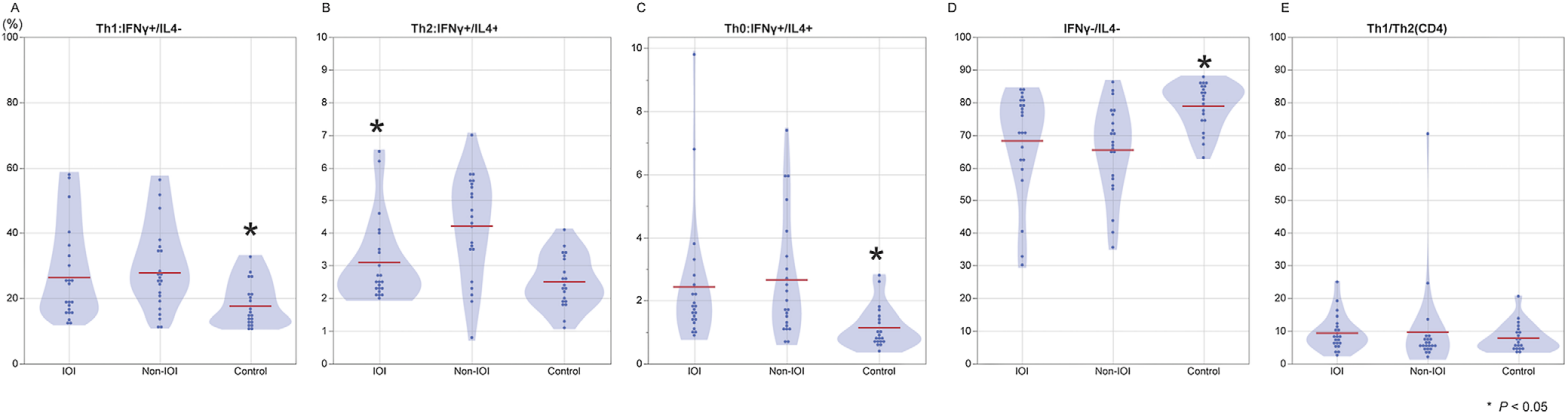
Fraction of CD4^+^ cells in the blood of the IOI, non-IOI, and control groups. There was a significant difference in the proportion of Th2 cells (IFN-γ/IL-4, 3.1% vs. 4.2%; *P* = 0.013) between IOI and non-IOI patients.

Figure 3 shows the percentages of IFN-γ- and IL-4-positive and IFN-γ- and IL-4-negative CD4^+^ cells in the serum and the Th1:Th2 ratios of the IOI and non-IOI groups. The percentage of Th2 cells was significantly lower in IOI patients than in non-IOI patients (3.1% vs. 4.2%, *P* = 0.013). As expected, after corrections were made for age and sex, as well as for age, sex, previous treatment, axial length, and posterior vitreous detachment, the results remained similar to those of the uncorrected results.

### Stepwise Variable Selection and ROC Curve to Estimate the Development of IOI

Following the identification of distinctively expressed factors between IOI and non-IOI patients, our group was motivated to develop an algorithm that could accurately assess the likelihood of IOI occurrence. To this end, we applied stepwise variable selection to determine the crucial factors in the aqueous humor, serum, and T-cell fraction in plasma, with and without serum biomarkers, that played a role in the development of IOI.

#### Aqueous Humor

**Figure 4.**
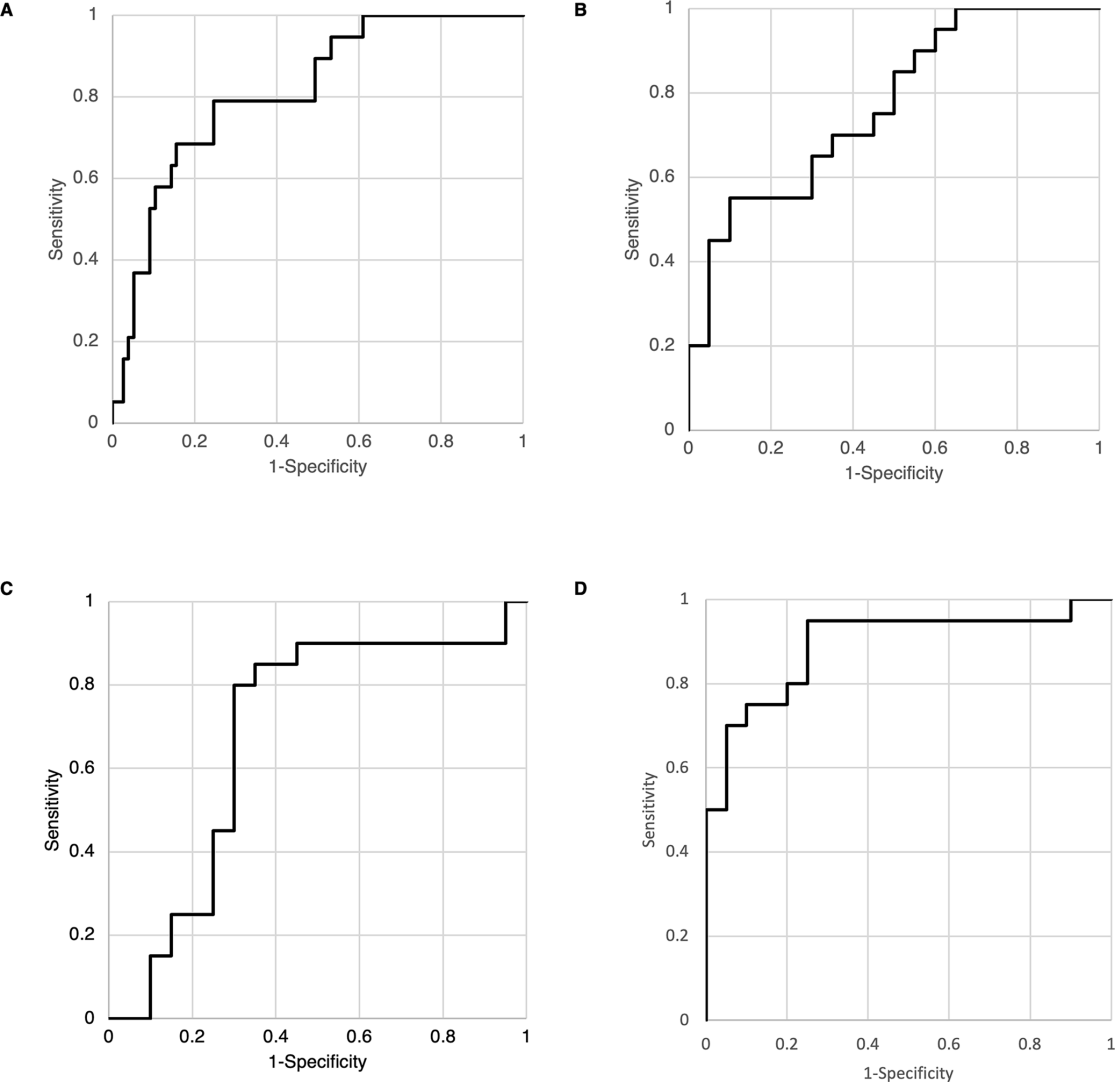
Area under the receiver operating characteristic curve. (**A**) Aqueous humor AUC for IOI estimation. Multivariate binary logistic regression analysis and the Youden index revealed that the equation −5.8 × log_10_(MCP-1) + 2.8 × log_10_(IP-10) + 2.6 × log_10_(P-selectin) + 2.0 × log_10_(CXCL1) + 1.1 × log_10_(VEGF-A) − 0.12 > 0 suggested the presence of IOI after brolucizumab injections. AUC, 0.81; sensitivity, 79%; and specificity, 75%. (**B**) Serum AUC for IOI estimation. Multivariate binary logistic regression analysis and the Youden index revealed that the equation −23 × log_10_(IL-6) + 3.2 × log_10_(MMP-9) + 19 × log_10_(IL-1β) − 8.1 > 0 suggested the presence of IOI after brolucizumab injections. AUC, 0.71; sensitivity, 70%; and specificity, 80%. (**C**) AUC for the fraction of CD4^+^ cells in the blood for IOI estimation. Th2 (*P* = 0.019) was selected by stepwise variable selection. Multivariate binary logistic regression analysis and the Youden index revealed that Th2 < 3.4 suggested the development of IOI after brolucizumab injections. AUC, 0.71; sensitivity, 73%; and specificity, 77%. (**D**) AUC for Th2 cells and serum protein concentrations. Multivariate binary logistic regression analysis and the Youden index revealed that the equation −1.2 × Th2 + 6.9 × log_10_(MMP-9) + 22 × log_10_(P-selectin) − 52 × log_10_(MCP-3) − 12 × log_10_(G-CSF) + 14 > 0 suggested the development of IOI after brolucizumab injections. AUC, 0.89; sensitivity, 95%; and specificity, 75%.

Using stepwise variable selection, we examined the discriminative ability of the cytokines for IOI occurrence via ROC analysis. Based on the cutoff value maximizing the Youden index, an optimal formula was derived (Fig. 4A):

$$\begin{array}{ccc} & & -5.8\times \mathrm{lo}g_{10}\left(\mathrm{MCP}-1\right)+2.8\times \mathrm{lo}g_{10}\left(\mathrm{IP}-10\right)\\ & & +2.6\times \log_{10}\left(P-\mathrm{selectin}\right)+2.0\times \log10\left(\mathrm{CXCL}1\right)\\ & & +1.1\times \log_{10}\left(\mathrm{VEGF}-A\right)-0.12>0 \end{array}$$

This yielded an AUC of 0.81 with a sensitivity of 79% and specificity of 75%.

#### Serum Biomarkers

Using the serum biomarkers, the following formula was able to discriminate patients who developed IOI after switching to brolucizumab from those who did not:

$$\begin{array}{ccc} & & -2.0\times \mathrm{lo}g_{10}\left(\mathrm{IL}-6\right)+0.86\times \mathrm{lo}g_{10}\left(\mathrm{MMP}-9\right)\\ & & +1.6\times \mathrm{lo}g_{10}\left(\mathrm{IL}-1\beta \right)-0.035>0 \end{array}$$

This yielded an AUC of 0.77 with a sensitivity of 55% and specificity of 10% (Fig. 4B).

#### T-Cell Fraction With or Without Serum Biomarkers

In the CD4^+^ cell fraction, Th2 (*P* = 0.019) was selected as a key variable by stepwise variable selection. Using a Youden index, a threshold Th2 value of 3.4 was determined to have high discriminative ability for the occurrence of IOI after switching to brolucizumab, with an AUC value of 0.71, sensitivity of 73%, and specificity of 23% (Fig. 4C).

Using the combination of the CD4^+^ cell fraction and serum protein concentrations, the following formula was estimated to predict IOI after switching to brolucizumab:

$$\begin{array}{ccc} & & -1.2\times \mathrm{Th}2+6.9\times \mathrm{lo}g_{10}\left(\mathrm{MMP}-9\right)+22\times \mathrm{lo}g_{10}\left(P-\mathrm{selectin}\right)\\ & & -52\times \mathrm{lo}g_{10}\left(\mathrm{MCP}-3\right)-12\times \mathrm{lo}g_{10}\left(G-\mathrm{CSF}\right)+14>0 \end{array}$$

This formula yielded an AUC of 0.89 with a sensitivity of 95% and specificity of 70% (Fig. 4D). Notably, no pair of variables had a correlation coefficient greater than 0.95.

## Discussion

In this study, IOI occurred 1 week to 10 months after the first intravitreal injection of brolucizumab. Onset was thus not immediately after injection, similar to previous reports,^5^ supporting the hypothesis that IOI is due to a delayed-type hypersensitivity.^6^

In a study examining the cause of IOI,^22^ it was speculated that the brolucizumab immune complexes formed by anti-drug antibodies (ADAs) against a non-native species of brolucizumab, resulting from the prolonged presence of brolucizumab at high concentrations in the eye, may cause a type III hypersensitivity reaction and trigger vasculitis. Also, brolucizumab immune complexes have been suggested to induce platelet aggregation and activate T cells, leading to type IV cell-mediated hypersensitivity reactions.^23^^–^^25^ Whereas type III hypersensitivity includes non-clearing complements that bind to excess antigen and is humoral and antigen dependent, type IV hypersensitivity involves cell-mediated immunity. Drug-hypersensitive CD4^+^ and CD8^+^ T cells are believed to be the major culprits for type IV delayed drug hypersensitivity reactions. In general, when sensitized memory T cells are restimulated by the same antigen, they induce cell-mediated immunity by producing cytokines, causing tissue damage and granuloma formation.^26^^,^^27^ The immune reaction involves cell-mediated immunity and therefore requires several days to activate lymphocytes. As such, this type of hypersensitivity is delayed. Traditionally, type IV hypersensitivity has been associated with the activity of CD4^+^ Th1 lymphocytes. Indeed, drug-specific T cells release various cytokines in a context-dependent manner. However, there are at least four main subtypes of type IV hypersensitivity,^28^^–^^30^ characterized by different combinations of inflammatory cells and cytokine profiles.

Brolucizumab-specific memory T and memory B cells are prerequisites for the development of IOI.^31^ Post-brolucizumab IOI is a complex process involving the formation of ADAs and subsequent immune reactions to ADAs and platelet aggregation. During this process, the ADA complex upregulates several cytokines from vascular endothelial cells (such as E-selectin, MMP-1, and integrin subunits β1 and 3) and peripheral blood mononuclear cells (such as TNF, IL-1β, and IL-6), which exacerbate the inflammation. Here, the IOI group had high levels of P-selectin, TNF-α, and IL-1α in the aqueous humor even before the initial brolucizumab injection. The combined altered expression of these cytokines may create an inflammatory environment that compromises the protective mechanisms promoting immune privilege in the posterior segment of the eye, thereby predisposing individuals to IOI development. This hypothesis is supported by the fact that a prior history of inflammation is a significant risk factor for developing IOI.^32^

The development of IOI may result in a Th1/Th2 cytokine imbalance. Compared with the non-IOI group, the IOI group had significantly higher serum MMP-9 levels and a lower proportion of plasma Th2 cells after the initial intravitreal injections of brolucizumab. MMP-9 is expressed predominantly in Th1 cells and participates in extracellular matrix remodeling and regulatory signaling during chronic inflammatory states. Analysis of the CD4^+^ cell fraction revealed that the proportion of Th2 cells was 25% smaller in patients who developed IOI than in non-IOI patients. We consider this to be a relatively large difference, considering the examples of other systemic diseases.^33^^,^^34^

Overall, our results suggest that a decreased proportion of Th2 cells is associated with IOI development, with an increase in the concentrations of MMP-9 triggered by the cytotoxicity of brolucizumab. Alternatively, it is also possible that the differences may have been present even before the brolucizumab injections. Further studies are needed to elucidate the roles of serum biomarkers in IOI.

Regarding the management of brolucizumab-associated IOI, local or systemic steroid administration is the only recommended treatment.^6^ One study reported 100% prevention of IOI by sub-Tenon's capsule triamcinolone acetonide injection.^35^ Immunosuppressants, such as tacrolimus, suppress T-cell–mediated immune responses and are used to lower the Th1-to-Th2 ratio, such as in fertility treatment.^36^ Tacrolimus is also used for general IOI and macular edema.^37^^–^^39^ We believe that it would be worthwhile to examine whether such immunosuppressant drugs would be useful in patients who require brolucizumab (e.g., nAMD patients in whom other drugs are ineffective) and patients with relative contraindications to steroids (e.g., steroid-induced glaucoma).

The lack of bilateral IOI after unilateral injection suggests that IOI results from a local immunological response. However, our results suggest that patients who are predisposed to IOI development may have unique T-cell populations, indicating that not only a local immunological response but also a systemic response may play a role in the occurrence of IOI, at least in part. Interestingly, when IOI developed after bilateral injection of brolucizumab, it occurred in both eyes without exception.

Because it is difficult to strictly control the conditions of each group in clinical studies, multivariate analysis is often used to control for the various factors. This may reveal hidden correlations or even eliminate pseudo-correlations. As a result, factors that were not significantly different in univariate analysis may become significantly different, or, conversely, they may not become significantly different, or the effects may even be reversed. Here, although external factors such as age and sex were matched, variable selection was also conducted to exclude confounding factors. Specifically, variance inflation factors (VIFs) serve as a diagnostic tool for detecting multicollinearity in regression analyses. When a VIF value exceeds 10, it indicates a high likelihood of multicollinearity, whereas values between 5 and 10 suggest a moderate degree of correlation among predictors. VIFs below 3 are generally interpreted as indicating the absence of multicollinearity.^40^^–^^42^ Notably, during the variable selection process, TNF-α and IL-1α were excluded due to their relatively high VIFs of 5.2 and 5.6, respectively. In multivariate analysis using all cytokine concentrations, IL-2 had a VIF of 10.0, which was another reason for its exclusion, although the difference was not significant in any analysis. Consequently, during the stepwise variable selection process, among the three variables showing statistically significance, only P-selectin remained, due to its lower VIF, which suggests less multicollinearity compared with the other variables.

There are some limitations to this study. First, it was a two-center study, and patient selection may thus be biased. Second, all of the patients were Japanese and had PCV, a specific subtype of nAMD. Therefore, further studies are needed to confirm whether our results are applicable to patients from other ethnic groups. Third, we were unable to obtain a validation cohort; however, because IOI does not occur at a particularly high rate, recruitment of a validation cohort is difficult. Fourth, we collected the blood samples at different time periods after IOI in each patient, which might have affected the measurements. Nevertheless, we believe that sufficient time had elapsed, that the systemic immune response to brolucizumab had resolved, and that the measured proteins and cell frequencies thus represented the baseline state. Fifth, we did not measure the serum concentrations of ADAs. Because the patients had prior anti-VEGF injections, we cannot rule out the possibility that they had been sensitized by structurally similar anti-VEGF drugs before switching. We are also aware of a recent report showing that an enhanced/boosted ADA may be associated with IOI.^43^ The central aim of the present study was to investigate the aqueous humor and serum protein profiles of patients with IOI, and our results indicate the importance of cell-mediated immune processes, in addition to such humoral- and antibody-dependent mechanisms. Sixth, it is also worth noting that sample selection could have introduced potential selection bias. In ROC analysis, sensitivity and specificity are susceptible to variation when applied to different populations, particularly when there are differences in the prevalence of IOI. The series of algorithms developed in this study will have to be validated in future work to establish their efficacy. Finally, a limitation of not applying Bonferroni’s correction in this study was that there was a higher likelihood of type I error. For many variables, it was assumed that the a posteriori Bonferroni’s correction would not reach statistical significance due to a small sample size, and an attempt was therefore made to apply a more conservative a posteriori analysis. This study was a descriptive correlational study, and our results do not indicate that the identified cytokines are discriminative biomarkers. Establishing these biomarkers and the efficacy of the algorithms will require validation in future work.

In conclusion, patients who developed IOI after intravitreal injections of brolucizumab had high P-selectin and IP-10 levels and low MCP-1 levels in the aqueous humor before switching. Our results suggest that high serum MMP-9 levels and a low proportion of plasma Th2 cells are discriminative biomarkers for IOI following intravitreal injection of brolucizumab. This was a descriptive correlational study, and our results do not indicate that the identified cytokines are discriminative biomarkers. Additionally, the blood samples were taken after injection. A validation cohort should be obtained to test the potential of these biomarkers to discriminate the occurrence of IOI. However, our findings do suggest mechanisms for brolucizumab-associated IOI. There may be an association between patients with Th2-dominant immunity and the occurrence of IOI after intravitreal brolucizumab injection.
